# Fresh Air in Railway Carriages

**Published:** 1910-09-10

**Authors:** D. G. Lawson

**Affiliations:** Assistant Secretary, Women's Imperial Health Association of Great Britain.


*To the Editor of* The Hospital.

Yours faithfully,

William J. Howarth, County M.O.H., Kent.

### FRESH AIR IN RAILWAY CARRIAGES.

*To the Editor of* The Hospital.

Dear Sir,—We were very pleased to see the remarks of Sir John Cockburn, President of the Congress of the Royal Sanitary Institute, opened at Brighton yesterday, with regard to the supply of fresh air in railway carriages, which is a subject that calls for the immediate attention of the authorities. A great deal of discomfort and train sickness, etc., which arises during railway travelling is undoubtedly caused by the vitiated air in which the travellers are compelled to sit. The legal right to insist upon the opening of a window has, we understand, never been definitely settled, and endeavours to assert it by some special air enthusiast have, we are sorry to say, not infrequently led to unseemly scenes in railway carriages. From every point of view, therefore, it is surely high time that the right to open the carriage window in certain carriages was definitely secured to insistent passengers. —Yours faithfully,

D. G. Lawson,
Assistant Secretary,
Women's Imperial Health Association of Great Britain.
September 6, 1910.

